# Cutaneous efficacy and glucocorticoid-sparing effects of anifrolumab in systemic lupus erythematosus: a systematic review and meta-analysis of randomized controlled trials

**DOI:** 10.3389/fimmu.2026.1910969

**Published:** 2026-09-30

**Authors:** Jianing Wang, Feiyin Liu, Qianwen Lou, Yinan Yang, Min Liu

**Affiliations:** School of Traditional Chinese Medicine, Beijing University of Chinese Medicine, Beijing, China

**Keywords:** anifrolumab, cutaneous response, glucocorticoid tapering, systemic lupus erythematosus, type I interferon

## Abstract

**Objective:**

To evaluate the cutaneous efficacy and glucocorticoid-sparing effects of anifrolumab in systemic lupus erythematosus (SLE) and to assess composite disease activity and flare-related and safety outcomes.

**Methods:**

PubMed, Embase, Web of Science, the Cochrane Library, and ClinicalTrials.gov were searched through 20 April 2026. Meta-analyses were restricted to randomized controlled trials comparing intravenous anifrolumab 300 mg every 4 weeks plus standard therapy with placebo plus standard therapy; TULIP-SC and overlapping reports were synthesized narratively. Risk ratios (RRs) or rate ratios with 95% confidence intervals (CIs) were pooled using random-effects models in the primary analyses, with fixed-effect models applied in sensitivity analyses. Modified Hartung-Knapp-Sidik-Jonkman (HKSJ) sensitivity analyses were performed for outcomes with substantial heterogeneity and a small number of contributing studies. Risk of bias and certainty of evidence were assessed using RoB 2 and GRADE, respectively.

**Results:**

In all, 12 reports from five independent trials were included; four intravenous trials contributed up to 1,203 participants. The Week 52 CLASI-A50 estimate was based on two trials (n = 165), not the full 1,203-participant IV evidence set. Random-effects pooling showed that anifrolumab improved Week 52 CLASI-A50 response based on two trials among patients with baseline CLASI-A ≥ 10 (RR 1.52, 95% CI 1.04 to 2.24), sustained oral glucocorticoid reduction in those receiving ≥ 10 mg/day at baseline (RR 1.56, 95% CI 1.30 to 1.87), and BICLA response (RR 1.66, 95% CI 1.41 to 1.94). SRI-4 (RR 1.39, 95% CI 1.04 to 1.87; I² = 80.8%), LLDAS attainment (RR 1.80, 95% CI 1.19 to 2.73; I² = 57.2%), and annualized flare rate (rate ratio 0.65, 95% CI 0.47 to 0.91; I² = 56.5%) favored anifrolumab but were affected by heterogeneity or limited trial numbers. The incidence of any adverse events was slightly higher (RR 1.09, 95% CI 1.03 to 1.16), whereas serious adverse events and discontinuations due to adverse events did not differ significantly. Supplementary analyses of selected adverse events of clinical interest suggested an increased risk of herpes zoster (RR 3.88, 95% CI 1.70 to 8.89), although event numbers were limited and these analyses were supplementary. Modified HKSJ sensitivity analyses yielded wider intervals crossing the null for SRI-4, LLDAS, annualized flare rate, serious adverse events, and adverse events leading to discontinuation.

**Conclusions:**

Intravenous anifrolumab improved Week 52 CLASI-A50 response in the two contributing trials of patients with baseline CLASI-A ≥ 10 and facilitated sustained glucocorticoid reduction in the prespecified glucocorticoid subgroup. DerSimonian-Laird point estimates for secondary efficacy outcomes generally favored anifrolumab, but the HKSJ sensitivity analyses for heterogeneous outcomes and the limited safety data warrant cautious interpretation.

**Systematic Review Registration:**

https://www.crd.york.ac.uk/PROSPERO/, identifier CRD420261367219.

## Introduction

Systemic lupus erythematosus (SLE) is a chronic and clinically heterogeneous autoimmune disease characterized by relapsing-remitting inflammation and multisystem involvement (1). Despite advances in background therapy, including antimalarials, glucocorticoids, immunosuppressants, and biologic agents (2), a substantial proportion of patients continue to experience active disease, recurrent flares, impaired quality of life, and cumulative organ damage (3, 4). These challenges underscore the need for targeted therapies that can improve disease control while reducing the long-term burden of treatment.

Cutaneous and mucocutaneous manifestations are common and clinically important features of SLE (5), and a large multinational cohort reported cumulative cutaneous activity in 36.5% of patients with SLE (6). Rash, alopecia, mucosal ulceration, and other lupus-related skin lesions can affect symptoms, body image, psychosocial wellbeing, and health-related quality of life (7). Cutaneous activity is also incorporated into composite disease activity measures and may reflect broader immunologic activation in some patients (8, 9). Improvement in skin-related outcomes is therefore an important therapeutic objective rather than an ancillary observation.

Although glucocorticoids remain important in the management of active SLE (10, 11), prolonged systemic exposure is associated with infections (12, 13), metabolic complications (14), osteoporosis (15), cardiovascular risk (16), and irreversible organ damage (17, 18). Contemporary SLE management therefore emphasizes minimizing glucocorticoid exposure while maintaining disease control (19, 20), making therapies that improve disease activity and facilitate sustained glucocorticoid reduction clinically important.

Type I interferon signaling contributes to immune activation, disease activity, and multiorgan manifestations in SLE (21, 22). It promotes sustained innate and adaptive immune activation and is particularly relevant to inflammatory cutaneous manifestations, making skin response an important clinical indicator of interferon-targeted treatment effects (11, 21). Anifrolumab targets subunit 1 of the type I interferon receptor and inhibits type I interferon signaling (23, 24), providing a biological rationale for targeting inflammatory disease activity, including skin involvement. Improved disease control through IFNAR1 blockade may also create conditions that permit protocol-guided glucocorticoid tapering. However, cutaneous improvement and glucocorticoid reduction are distinct clinical outcomes, and the available trial-level evidence cannot determine whether improvement in skin disease directly contributes to glucocorticoid reduction. Randomized trials, including MUSE (23), TULIP-1 (25), TULIP-2 (26), TULIP-SC (27), and an Asian phase III study (28), provide the main randomized evidence base for evaluating these outcomes.

Previous meta-analyses have evaluated the overall efficacy, safety, or broad organ-domain effects of anifrolumab, and some have also pooled cutaneous responses and glucocorticoid-tapering outcomes (22, 29, 30). However, these outcomes were generally embedded within broader efficacy frameworks and were not consistently interpreted in relation to their distinct prespecified baseline populations. The handling of intravenous and subcutaneous regimens and overlapping reports from the same parent trials has also varied across reviews. This systematic review and meta-analysis therefore jointly prioritized cutaneous response and sustained glucocorticoid reduction as two principal clinical outcomes, explicitly linked each estimate to its applicable baseline population, and incorporated the publicly reported Asian phase III results, while also examining BICLA response, SRI-4 response, flare-related outcomes, and safety.

Because several eligible reports were derived from overlapping trial populations or evaluated a different route of administration, quantitative pooling was limited to the four intravenous trials, while TULIP-SC and supplementary reports were described separately.

## Methods

### Compliance with preferred reporting items for systematic reviews and meta-analyses guidelines

This study was conducted and reported in accordance with the PRISMA 2020 statement (31), and the protocol was registered in PROSPERO (CRD420261367219). Cutaneous efficacy and glucocorticoid-sparing effects were prespecified as the primary outcomes. The synthesis plan distinguished the primary IV meta-analysis from the single TULIP-SC subcutaneous trial and overlapping supplementary reports.

### Eligibility criteria

#### Inclusion criteria

Eligible studies were randomized controlled trials enrolling adults with systemic lupus erythematosus diagnosed or classified according to internationally recognized criteria, including the 2019 EULAR/ACR, 2012 SLICC, or 1997 ACR criteria (32–34). Studies evaluating anifrolumab in adult SLE were eligible for the systematic review; the primary quantitative synthesis was restricted to the approved IV regimen of anifrolumab 300 mg every 4 weeks plus standard therapy versus placebo plus standard therapy (35). Trials of other routes or regimens, including subcutaneous anifrolumab, were summarized when relevant but were not pooled with the IV trials.

Studies had to report at least one extractable outcome related to cutaneous efficacy, glucocorticoid-sparing effects, composite disease activity, flares, or safety. Eligible outcomes included CLASI-A activity score change, CLASI-A50 or related mucocutaneous measures, sustained oral glucocorticoid reduction to ≤7.5 mg/day from Week 40 through Week 52 among patients receiving ≥10 mg/day at baseline, BICLA response, SRI-4 response, LLDAS attainment, DORIS remission, annualized flare rate, any adverse events, serious adverse events, infections, herpes zoster, death, injection-site reactions or infusion-related reactions, and adverse events leading to discontinuation. Eligibility was determined at the trial level. Full-text articles were used preferentially; registry results were eligible when sufficiently detailed. Conference abstracts, appendices, and subgroup or *post hoc* reports were used only to supplement eligible parent trials or provide narrative context.

#### Exclusion criteria

Studies were excluded if they did not enroll patients with SLE, did not evaluate anifrolumab, were not randomized controlled trials, lacked extractable outcomes, or were reviews, guidelines, commentaries, editorials, letters, observational studies, case reports, animal studies, or *in vitro* studies. Duplicate or overlapping reports were not treated as independent evidence sources; the original full-text trial report was used preferentially, with secondary reports used only for supplementary outcome data. Pooled or *post hoc* TULIP reports were not treated as additional independent trials. When individual trial-level data were unavailable, such sources were used only as supplementary or exploratory evidence-source syntheses and were explicitly identified as overlapping TULIP-derived data. Studies of non-SLE populations or overlap syndromes were excluded when SLE-specific data could not be extracted.

### Information sources and search strategy

Two reviewers searched PubMed, Embase, Web of Science, the Cochrane Library, and ClinicalTrials.gov from 1 January 2011 to 20 April 2026. Search strategies combined terms for SLE, anifrolumab/MEDI-546/Saphnelo, and randomized trials; complete strategies are provided in online **Supplementary Table S1**. Relevant reviews, meta-analyses, reference lists, conference abstracts, **Supplementary Materials**, and sponsor trial-result materials were also screened. FDA and EMA regulatory databases were not searched as independent sources.

### Study selection

Two reviewers independently screened titles, abstracts, and full texts after duplicates were removed. Exclusion reasons were recorded when applicable. Disagreements were resolved by discussion or adjudication by a third reviewer.

### Data extraction

Two reviewers independently extracted data using a standardized form. Extracted items included study design, trial phase, country or region, follow-up duration, route and dose, sample size, baseline characteristics, baseline CLASI-A score and oral glucocorticoid use, outcome definitions, time points, event counts, denominators, and rate-based summary estimates. Week 52 data were preferentially extracted. Although the protocol included CLASI-A activity score change as a cutaneous outcome, continuous change data were not reported consistently in a form suitable for quantitative pooling across trials. Therefore, the quantitative cutaneous synthesis used CLASI-A50 responder data, restricted to the baseline CLASI-A eligibility subgroup reported by the source trials. Primary efficacy outcomes were CLASI-A50 response and glucocorticoid dose reduction; secondary efficacy outcomes were BICLA response, SRI-4 response, and annualized flare rate. LLDAS was assessed in an exploratory evidence-source synthesis when only pooled/*post hoc* TULIP-derived data were extractable. Quantitative safety outcomes were any adverse events, serious adverse events, and discontinuations due to adverse events, with selected adverse events of clinical interest summarized in supplementary analyses.

Definitions of CLASI-A50 (23, 36), BICLA (23, 37), and SRI-4 (23, 38) were based on validated instruments and the prespecified responder criteria in the source trials. Efficacy data were extracted from the primary efficacy analysis set or the prespecified subgroup; safety data were extracted from the safety analysis set. For CLASI-A50, data were restricted to patients meeting the baseline CLASI-A threshold in the source trial; this subgroup restriction was source-defined and should not be generalized to all randomized SLE participants. For glucocorticoid reduction, data were restricted to patients receiving ≥10 mg/day at baseline. Arm-level counts and denominators were extracted directly when available. Adjusted, weighted, model-based, or posterior response rates were not converted into event counts. For annualized flare rate, model-based rate ratios and 95% CIs were extracted from the source studies. Trial-specific definitions of background standard therapy, restricted medications, rescue therapy, glucocorticoid tapering windows, and response rules were followed and recorded as potential sources of methodological heterogeneity. Detailed outcome definitions and data extraction rules are provided in online **Supplementary Table S4**.

### Risk of bias assessment

Two reviewers independently assessed risk of bias using the Cochrane RoB 2 tool (39, 40). Five domains were evaluated: randomization, deviations from intended interventions, missing outcome data, outcome measurement, and selection of the reported result. Each domain and the overall judgment were rated as low risk, some concerns, or high risk. Disagreements were resolved by discussion or adjudication by a third reviewer, and results were visualized using Review Manager 5.4.

### Certainty of evidence assessment

Certainty of evidence was assessed using the GRADE approach for primary efficacy outcomes, secondary efficacy outcomes, and pooled safety outcomes (41). Randomized evidence started as high certainty and could be downgraded for risk of bias, inconsistency, indirectness, imprecision, or publication bias. Downgrading decisions considered RoB 2 judgments, I², direction and overlap of effects, alignment of population and outcome definitions with the review question, event numbers, sample size, and the width of the 95% CIs. Because fewer than 10 studies were included in each quantitative synthesis, publication bias was assessed qualitatively using search completeness, outcome reporting, and reporting source type, following guidance on risk of bias due to missing evidence (42). A summary of findings table was generated using GRADEpro GDT.

### Statistical analysis

Analyses were performed in R version 4.5.1, primarily using the meta package. Dichotomous outcomes were summarized as risk ratios (RRs) with 95% CIs and pooled using the Mantel-Haenszel method. Annualized flare rate was pooled as a rate ratio using the generic inverse-variance method. When a standard error was unavailable, it was calculated as SE = [ln(upper 95% CI) − ln(lower 95% CI)]/3.92, and pooled estimates were back-transformed. All statistical tests were two-sided, and P < 0.05 was considered statistically significant.

All primary quantitative meta-analyses used random-effects models with the DerSimonian-Laird estimator to account for anticipated clinical and methodological heterogeneity across trials. Dichotomous outcomes were pooled using Mantel-Haenszel random-effects models, and annualized flare rate was pooled using a generic inverse-variance random-effects model. Fixed-effect models were used as sensitivity analyses to assess robustness to model choice. Heterogeneity was assessed using τ², I², and Cochran’s Q test.

The primary meta-analysis was restricted to IV anifrolumab 300 mg every 4 weeks. TULIP-SC was summarized narratively. Outcomes that could not be pooled because of inconsistent reporting, overlapping sources, route-specific reporting, or sparse events were summarized narratively or in supplementary analyses. LLDAS was retained only as an exploratory evidence-source synthesis because the available extractable data combined MUSE with a pooled TULIP-1/TULIP-2 *post hoc* source; DORIS remission was summarized narratively because available data came mainly from overlapping *post hoc* or long-term extension sources (43).

Additional sensitivity analyses assessed Week 12 CLASI-A50 and SRI-4 after excluding TULIP-1. Sensitivity analyses excluding the registry-only AZALEA Asian phase III trial (NCT04931563) were performed for sustained oral glucocorticoid reduction, BICLA response, and annualized flare rate, because this trial contributed materially to these pooled estimates. For outcomes with substantial heterogeneity and a small number of contributing studies, modified Hartung-Knapp-Sidik-Jonkman (HKSJ) sensitivity analyses were performed using study-level log risk ratios or log rate ratios. These analyses assessed uncertainty around conventional DerSimonian-Laird random-effects confidence intervals and did not replace the primary random-effects analyses; results are provided in online **Supplementary Table S6**. Funnel plots and formal tests for funnel-plot asymmetry were not performed because fewer than 10 studies contributed to each synthesis (44).

Deviations from the PROSPERO protocol, including changes to the primary statistical model and outcome handling because of data availability, are summarized with rationales in **Supplementary Table S7**.

## Results

### Literature search

Database and registry searches identified 1,075 records, including 1,039 from electronic databases and 36 from trial registries. After removing 477 duplicates, 598 records underwent title and abstract screening; 526 were excluded as irrelevant or ineligible. A total of 72 reports were assessed in full text, and 60 were excluded because they did not meet eligibility criteria (n = 35), lacked posted or extractable trial results or were ongoing studies (n = 16), duplicated or overlapped with included data (n = 5), or were non-randomized or observational studies, preprint, commentary, mechanistic reports, or incompatible extension reports (n = 4).

A total of 12 reports from five independent randomized controlled trials were included. Four IV anifrolumab 300 mg every 4 weeks trials contributed to the primary quantitative synthesis. TULIP-SC was summarized narratively, and seven overlapping pooled, *post hoc*, subgroup, or long-term reports provided additional contextual outcome data. The selection process is shown in **Figure 1**, and the evidence structure is shown in **Figure 2**.

**Figure 1:**
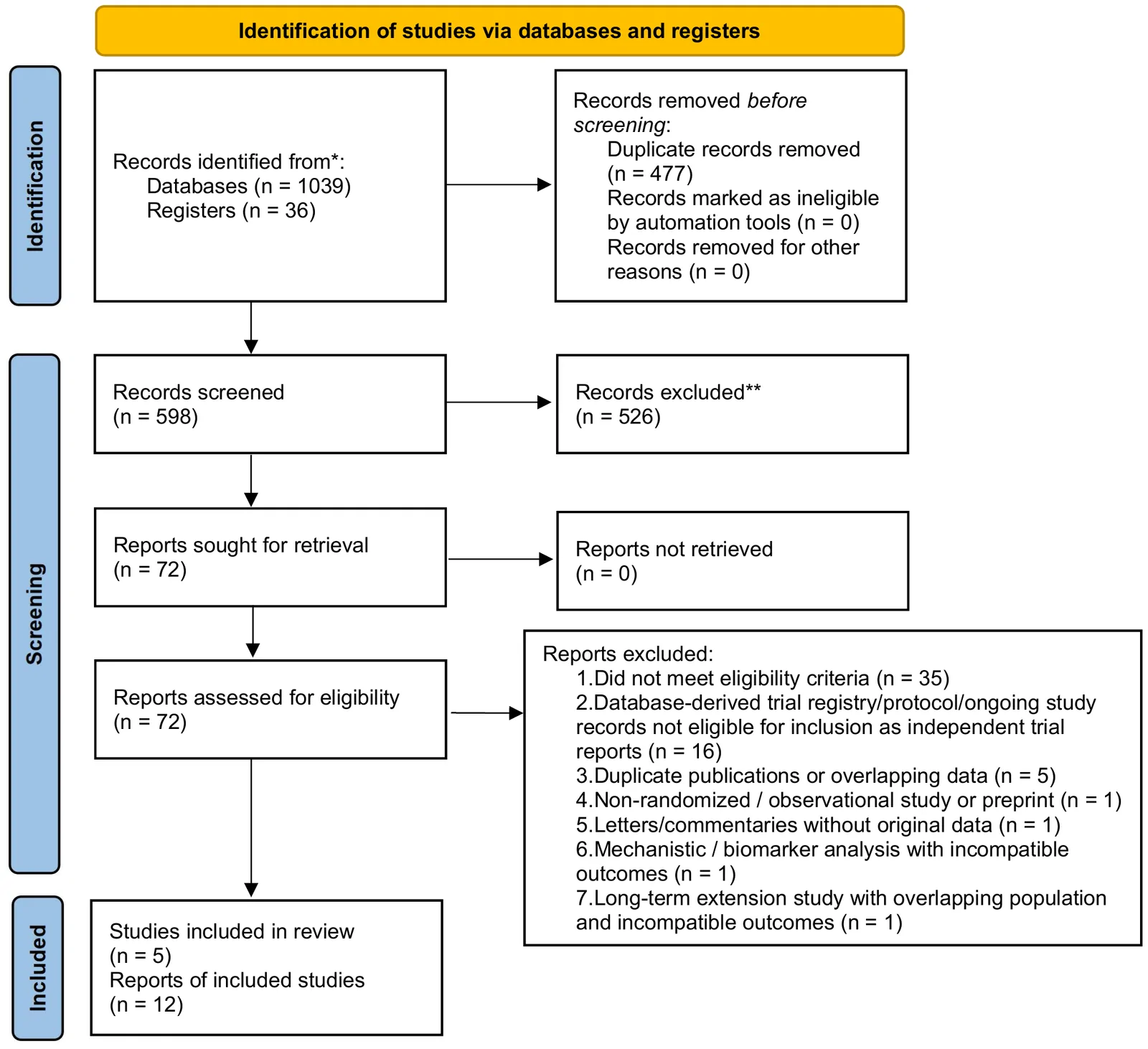
PRISMA flow diagram of study selection.

**Figure 2:**
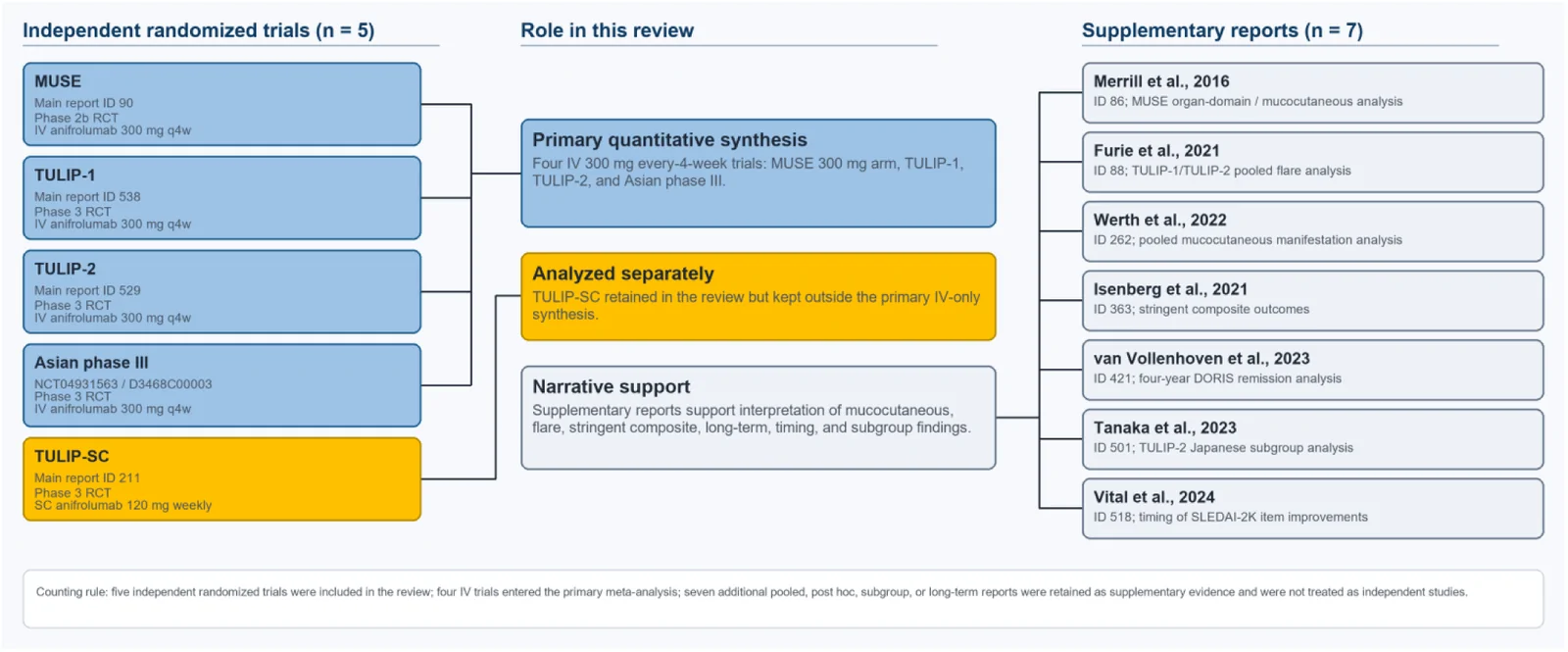
Evidence structure of included trials and supplementary reports.

### Characteristics of included studies

The included trials are summarized in **Table 1**. Five independent randomized, double-blind, placebo-controlled trials evaluated anifrolumab in adults with active SLE: MUSE, TULIP-1, TULIP-2, the Asian phase III trial, and TULIP-SC. Four trials evaluated IV anifrolumab 300 mg every 4 weeks plus standard therapy; TULIP-SC evaluated SC anifrolumab 120 mg weekly and is described separately.

**Table 1:** Characteristics of the included randomized controlled trials.

Study	Report source	Study design	Phase	Sample size	Follow-up duration	Outcomes reported	
						Efficacy	Safety
MUSE (23)	Furie et al., 2017, Arthritis & Rheumatology	Multicenter RCT	Phase IIb	N = 201 for the main comparison (99 vs 102); total mITT population = 305.	Treatment through Week 48; efficacy assessed at Weeks 24 and 52, followed by a 12-week follow-up period.	①②③④⑤	⑦⑧⑨
TULIP-1 (25)	Furie et al., 2019, Lancet Rheumatology	Multicenter RCT	Phase III	N = 364 for the main comparison (180 vs 184); total randomized population = 457.	Treatment through Week 48; final assessment at Week 52.	①②③④⑥	⑦⑧⑨
TULIP-2 (26)	Morand et al., 2020, New England Journal of Medicine	Multicenter RCT	Phase III	N = 362 (180 vs 182).	Treatment through Week 48; primary endpoint assessed at Week 52.	①②③④⑥	⑦⑧⑨
Asian phase III (D3468C00003/NCT04931563)	ClinicalTrials.gov registry results (NCT04931563; D3468C00003) (28)	Multicenter RCT	Phase III	N = 276 in the available results set (138 vs 138); actual enrollment = 277.	Treatment from Week 0 to Week 48; primary endpoint assessed at Week 52.	②③④⑥	⑧⑨
TULIP-SC (27)	Manzi et al., 2026, Arthritis & Rheumatology	Multicenter RCT	Phase III	N = 367 in the full analysis set (184 vs 183); interim analysis = 220.	52-week double-blind treatment period; Week 52 data reported.	①*②③④⑥	⑧⑨

Across comparison groups, the five trials contributed 1,570 participants. The four IV trials contributed up to 1,203 participants to the primary quantitative synthesis, whereas TULIP-SC contributed 367 participants. Denominators differed by outcome: Week 52 CLASI-A50 was restricted to patients with baseline CLASI-A ≥10 (n = 165); glucocorticoid reduction, to patients with baseline OCS ≥10 mg/day (n = 679); BICLA, SRI-4, and major safety outcomes, to approximately 1,202–1,203 participants; selected adverse-event outcomes, to the trials or reports with available outcome-specific data; and LLDAS, to MUSE plus a pooled TULIP-1/TULIP-2 *post hoc* source. The LLDAS estimate was therefore treated as exploratory.

Values in parentheses indicate the number of patients in the anifrolumab and placebo groups, respectively. mITT, modified intention-to-treat.

Outcome codes: ① CLASI-A50 response; ② sustained oral glucocorticoid reduction; ③ BICLA response; ④ SRI-4 response; ⑤ LLDAS attainment; ⑥ annualized flare rate; ⑦ any adverse events; ⑧ serious adverse events; ⑨ discontinuations due to adverse events.

*TULIP-SC reported *post hoc* CLASI-50/CLASI-A50-related cutaneous outcomes and was summarized narratively; it was not included in the primary intravenous meta-analysis.

### Risk of bias assessment

Risk-of-bias judgments are shown in **Figure 3** and online **Supplementary Table S3**. No trial was judged to be at high risk of bias. TULIP-1 and TULIP-2 were rated low risk across all RoB 2 domains. MUSE, the Asian phase III trial, and TULIP-SC were judged to have some concerns, mainly because of possible selection of the reported result; the registry-based Asian phase III trial also had incomplete public information on randomization and missing outcome data. All trials were judged to be at low risk for deviations from intended interventions and outcome measurement.

**Figure 3:**
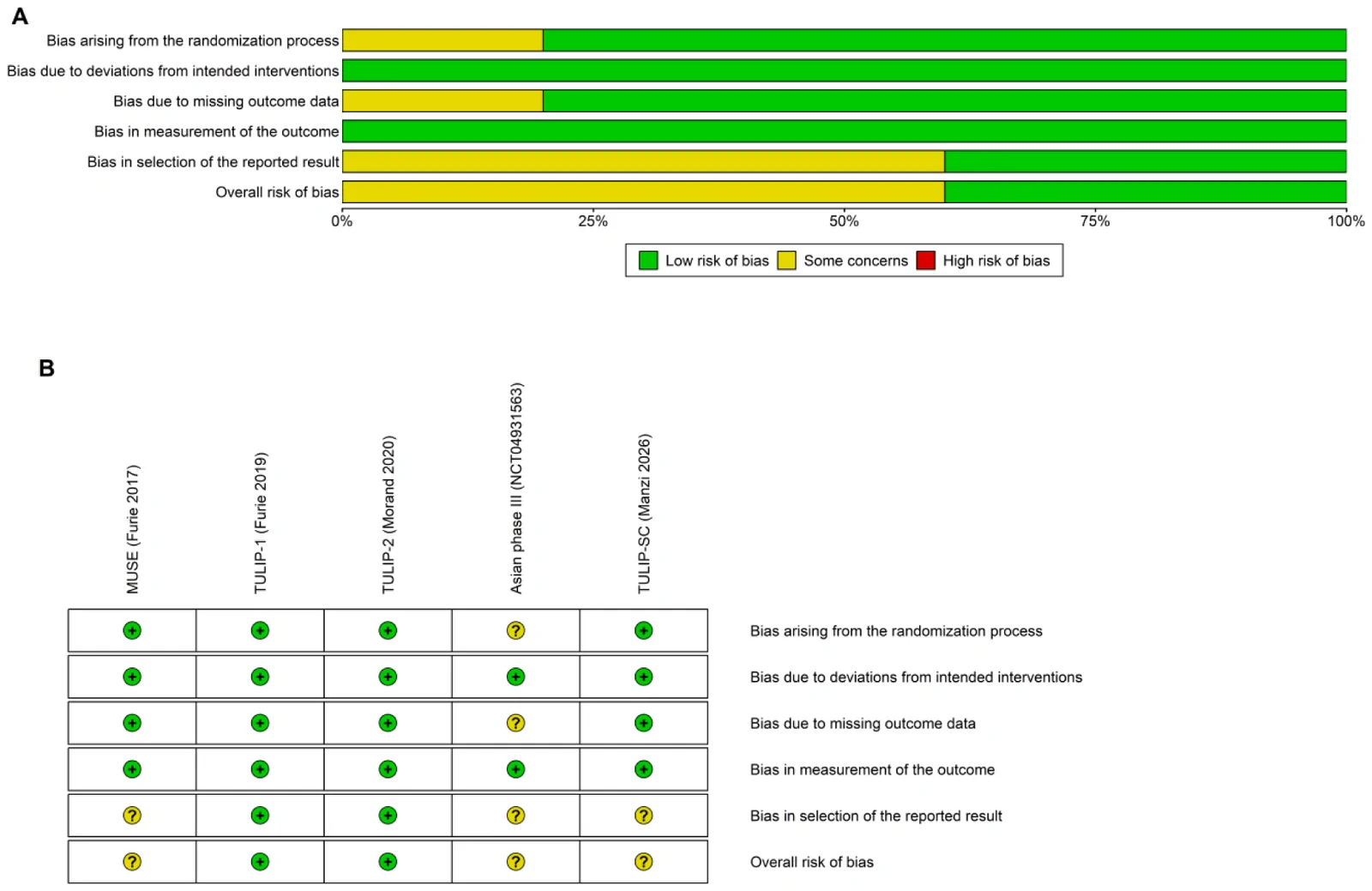
Risk-of-bias assessment of the included randomized controlled trials. **(A)** Risk-of-bias summary across studies. **(B)** Domain-level risk-of-bias judgments for each study.

### Summary of pooled efficacy and safety outcomes

**Table 2** provides a compact summary of the primary IV quantitative synthesis based on random-effects models, with LLDAS explicitly identified as an exploratory evidence-source synthesis because it incorporated a pooled TULIP-1/TULIP-2 *post hoc* source. The following sections report the two prespecified primary outcomes in detail and present secondary efficacy and pooled safety outcomes more selectively, with full numerical details shown in **Table 2** and **Figures 4**–**6**. Selected adverse events of clinical interest, fixed-effect sensitivity analyses, and the sensitivity analysis excluding the AZALEA Asian phase III trial are shown in **Supplementary Figures S1** and **S3–S8**; HKSJ sensitivity analyses are shown in online **Supplementary Table S6**. TULIP-SC and overlapping supplementary reports are described separately.

**Table 2:** Summary of pooled efficacy and safety outcomes.

Outcome	Studies included	Analysis set	Effect measure/model	Pooled effect, 95% CI	I² (%)	Interpretation
CLASI-A50 response	2	Baseline CLASI-A ≥10	RR; MH random-effects	RR = 1.52 (95% CI 1.04–2.24)	19.5	Favored anifrolumab among patients with baseline CLASI-A ≥10
Sustained oral glucocorticoid reduction	4	Baseline OCS ≥10 mg/day	RR; MH random-effects	RR = 1.56 (95% CI 1.30–1.87)	9.3	Favored anifrolumab in the baseline OCS ≥10 mg/day subgroup
BICLA response	4	mITT population	RR; MH random-effects	RR = 1.66 (95% CI 1.41–1.94)	12.8	Favored anifrolumab
SRI-4 response	4	mITT population	RR; MH random-effects	RR = 1.39 (95% CI 1.04–1.87)	80.8	Favored anifrolumab, with substantial heterogeneity. Modified HKSJ CI crossed 1, indicating sensitivity to small-study random-effects uncertainty.
LLDAS attainment	2	Eligible IV evidence sources	RR; MH random-effects	RR = 1.80 (95% CI 1.19–2.73)	57.2	Favored anifrolumab in an exploratory evidence-source synthesis; not a conventional independent trial-level meta-analysis. Modified HKSJ CI was extremely wide and crossed 1; supportive only.
Annualized flare rate	3	Trials reporting rate ratios	Rate ratio; GIV random-effects	Rate ratio = 0.65 (95% CI 0.47–0.91)	56.5	Favored anifrolumab; limited by three trials and moderate heterogeneity. Modified HKSJ CI crossed 1; interpret as supportive.
Any adverse events	3	Safety population	RR; MH random-effects	RR = 1.09 (95% CI 1.03–1.16)	0.0	Slightly increased with anifrolumab
Serious adverse events	4	Safety population	RR; MH random-effects	RR = 0.90 (95% CI 0.54–1.50)	70.4	No statistically significant difference; wide CI and substantial heterogeneity. Modified HKSJ CI was wider and crossed 1, reinforcing uncertainty.
Discontinuations due to adverse events	4	Safety population	RR; MH random-effects	RR = 0.91 (95% CI 0.33–2.55)	66.0	No statistically significant difference; wide CI and substantial heterogeneity. Modified HKSJ CI was very wide and crossed 1, reinforcing uncertainty due to sparse events.

The LLDAS estimate combines MUSE with a pooled TULIP-1/TULIP-2 *post hoc* source and should not be interpreted as a conventional independent trial-level meta-analysis.

**Figure 4:**
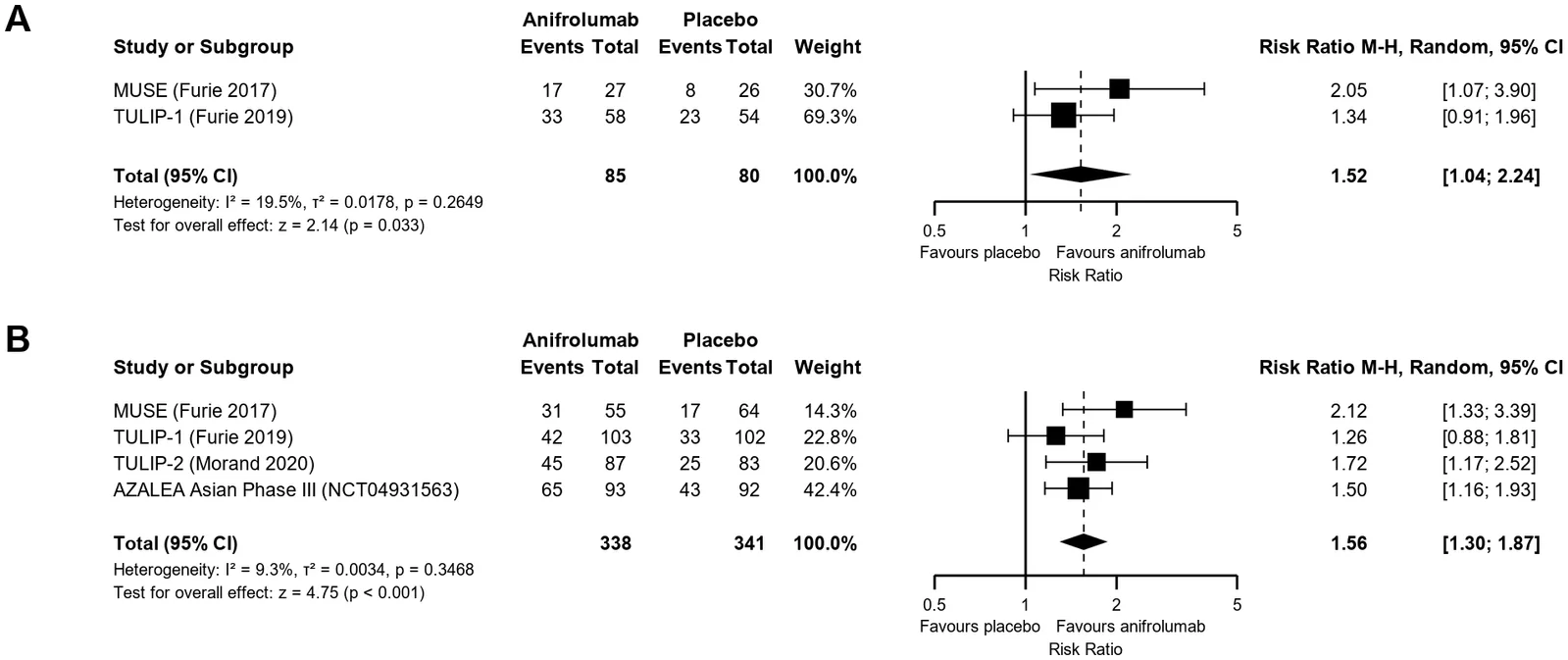
Primary efficacy outcomes. **(A)** CLASI-A50 response at Week 52. **(B)** Sustained oral glucocorticoid dose reduction.

**Figure 5:**
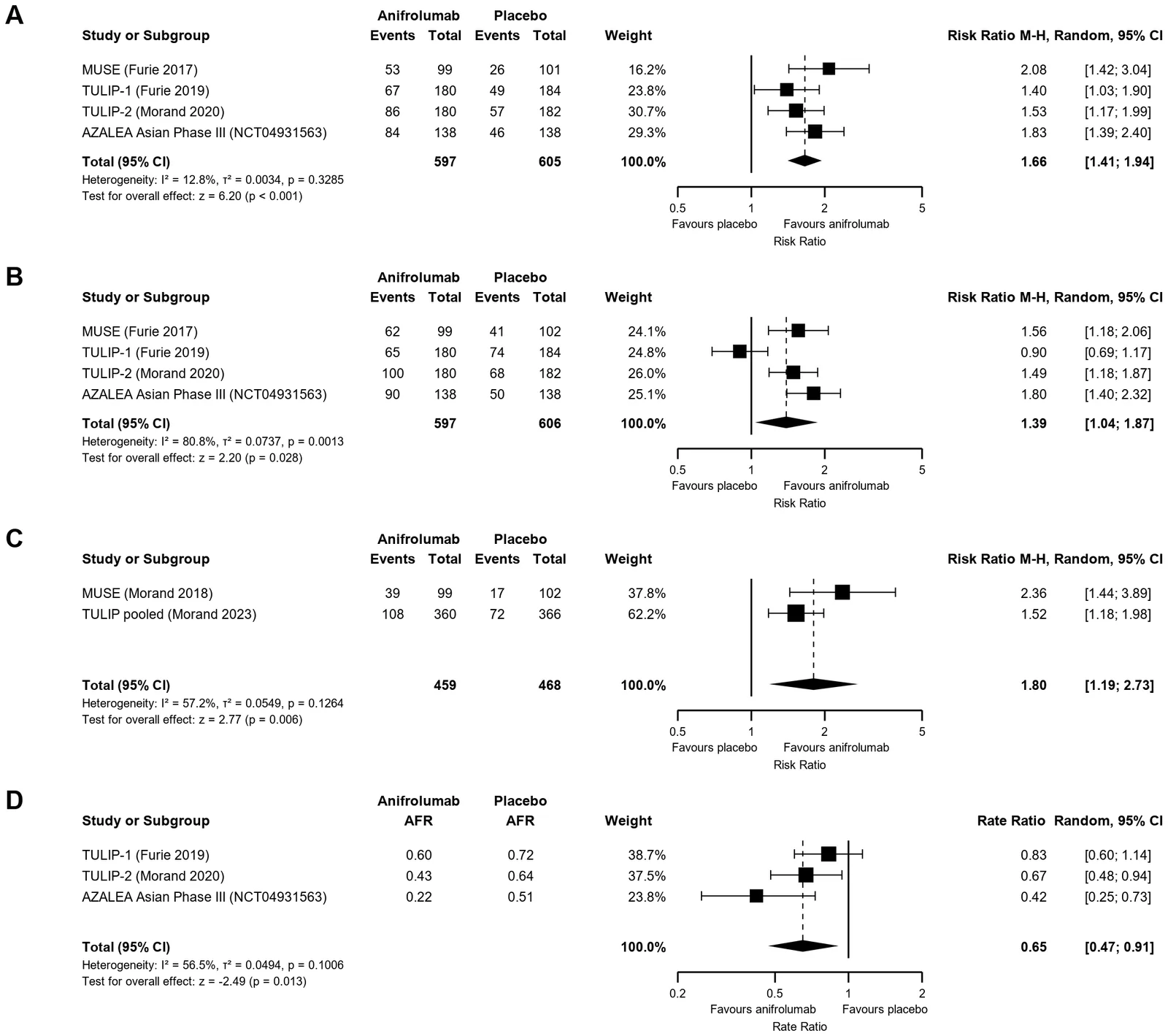
Secondary efficacy outcomes. **(A)** BICLA response at Week 52. **(B)** SRI-4 response at Week 52. **(C)** LLDAS attainment at Week 52. **(D)** Annualized flare rate.

**Figure 6:**
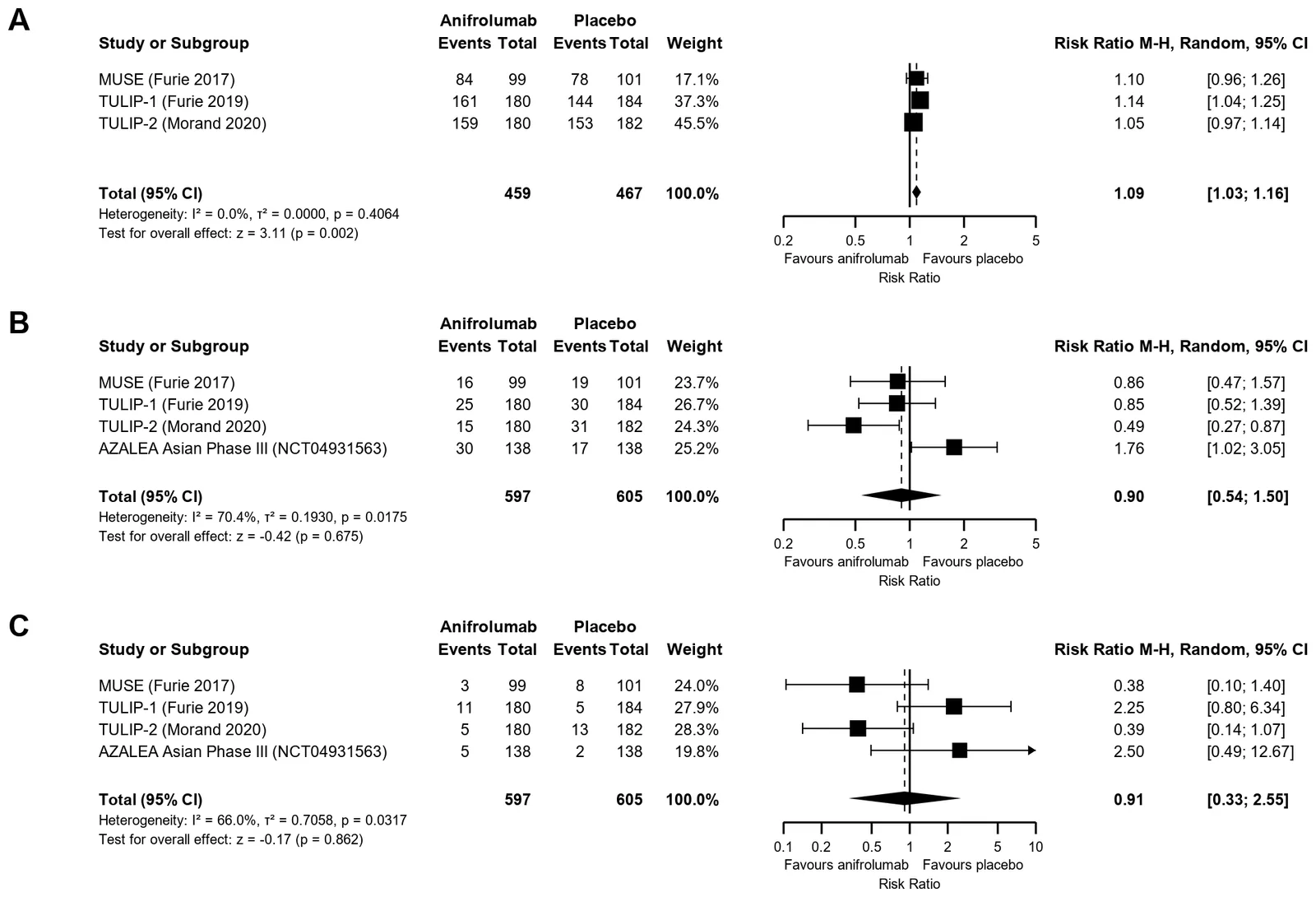
Safety outcomes. **(A)** Any adverse events. **(B)** Serious adverse events. **(C)** Adverse events leading to discontinuation.

RR, risk ratio; MH, Mantel–Haenszel; GIV, generic inverse variance; CI, confidence interval; mITT, modified intention-to-treat; OCS, oral corticosteroid; AEs, adverse events; LLDAS, lupus low disease activity state; DL, DerSimonian-Laird; HKSJ, Hartung-Knapp-Sidik-Jonkman.

For efficacy outcomes, RR > 1 favored anifrolumab, whereas for annualized flare rate, a rate ratio < 1 favored anifrolumab. For safety outcomes, RR > 1 indicated increased risk. Statistical significance was determined by whether the 95% CI crossed 1.

### Primary efficacy outcomes

#### CLASI-A50

Week 52 CLASI-A50 response was reported in two trials among patients with baseline CLASI-A ≥10. TULIP-2 reported CLASI-A50 at Week 12 in this baseline CLASI-A ≥10 subgroup, but Week 52 CLASI-A50 responder counts were not reported in an extractable form; therefore, TULIP-2 was not included in the Week 52 primary cutaneous synthesis. This synthesis therefore represents a source-defined active cutaneous subgroup rather than the overall randomized SLE population.

More patients achieved CLASI-A50 with anifrolumab than with placebo (50/85, 58.8% vs 31/80, 38.8%). Random-effects pooling showed a benefit with anifrolumab (RR = 1.52, 95% CI 1.04–2.24, P = 0.033), with low observed heterogeneity, although the heterogeneity estimate had limited diagnostic value because only two trials contributed (I² = 19.5%) (**Figure 4A**). Because only two trials contributed to this analysis, the I² estimate should be interpreted cautiously, as the small number of studies limits the precision of between-study heterogeneity estimation.

An exploratory Week 12 analysis using TULIP-1 and TULIP-2 also favored anifrolumab (RR = 1.75, 95% CI 1.17–2.62, P = 0.007; I² = 0%) (**Supplementary Figure S2A**). No statistical heterogeneity was detected in this exploratory Week 12 analysis; however, the I² value should be interpreted cautiously because only two trials contributed data. With such a small number of studies, I² has limited ability to characterize between-study heterogeneity. Because the Week 12 and Week 52 analyses included different trial sets, the Week 12 result was interpreted as supportive rather than as direct evidence of temporal consistency.

#### Glucocorticoid dose reduction

Sustained oral glucocorticoid reduction was reported in four trials among patients receiving ≥10 mg/day at baseline and was defined as a reduction to ≤7.5 mg/day maintained from Week 40 through Week 52. More patients achieved sustained reduction with anifrolumab than with placebo (183/338, 54.1% vs 118/341, 34.6%). Random-effects pooling showed a significant benefit (RR = 1.56, 95% CI 1.30–1.87, P < 0.001), with low observed heterogeneity (I² = 9.3%) (**Figure 4B**). In a sensitivity analysis excluding the registry-only AZALEA Asian phase III trial (NCT04931563), the pooled effect remained statistically significant and favored anifrolumab (RR = 1.62, 95% CI 1.21–2.17, P = 0.001; I² = 36.8%) (**Supplementary Figure S8**). This result applies only to the prespecified subgroup receiving oral glucocorticoids at ≥10 mg/day at baseline.

### Secondary efficacy outcomes

#### BICLA response

BICLA response was reported in four trials; random-effects pooling favored anifrolumab (RR = 1.66, 95% CI 1.41–1.94; I² = 12.8%; **Figure 5A**; **Table 2**).

#### SRI-4 response

SRI-4 response was reported in four trials; random-effects pooling favored anifrolumab (RR = 1.39, 95% CI 1.04–1.87; I² = 80.8%; **Figure 5B**; **Table 2**). Heterogeneity and small-study random-effects uncertainty were the main limitations. The sensitivity analysis excluding TULIP-1 reduced heterogeneity and preserved the direction of effect, identifying a trial-level source of inconsistency without replacing the prespecified all-trial analysis (**Supplementary Figure S2B**).

#### LLDAS attainment

LLDAS attainment was evaluated as an exploratory evidence-source synthesis using two IV evidence sources: MUSE and a pooled TULIP-1/TULIP-2 *post hoc* source. Random-effects pooling favored anifrolumab (RR = 1.80, 95% CI 1.19–2.73; I² = 57.2%; **Figure 5C**; **Table 2**), but this estimate should not be interpreted as an independent trial-level meta-analysis. Because this outcome was derived from limited contributing sources and showed moderate heterogeneity, it was interpreted as supportive rather than definitive evidence. The very wide interval from the modified HKSJ analysis further supports treating LLDAS as supportive rather than confirmatory evidence.

#### Annualized flare rate

Annualized flare rate was reported in three trials. Random-effects pooling favored anifrolumab (rate ratio = 0.65, 95% CI 0.47–0.91; I² = 56.5%; **Figure 5D**; **Table 2**). Certainty was limited by the small number of trials and heterogeneity.

### Safety outcomes

The quantitative safety synthesis included any adverse events, serious adverse events, and discontinuations due to adverse events. Selected adverse events of clinical interest, including upper respiratory tract infection, herpes zoster, bronchitis, and infusion-related reactions, were summarized in supplementary random-effects analyses. Deaths and injection-site reactions were not treated as primary pooled endpoints because events or route-specific reports were sparse and were more appropriately summarized narratively where available.

#### Any adverse events

Data on any adverse events were reported in three trials. Random-effects pooling showed a modest increase with anifrolumab (RR = 1.09, 95% CI 1.03–1.16; I² = 0%; **Figure 6A**; **Table 2**).

#### Serious adverse events

Serious adverse events were reported in four trials. Random-effects pooling did not show a statistically significant difference (RR = 0.90, 95% CI 0.54–1.50; I² = 70.4%; **Figure 6B**; **Table 2**). The confidence interval and between-study variability leave uncertainty about the direction and magnitude of any serious adverse-event difference.

#### Discontinuations due to adverse events

Discontinuations due to adverse events were reported in four trials. Random-effects pooling did not show a statistically significant difference (RR = 0.91, 95% CI 0.33–2.55; I² = 66.0%; **Figure 6C**; **Table 2**). Few events, wide confidence intervals, and between-study variability limit interpretation.

#### Selected adverse events of clinical interest

Supplementary random-effects analyses suggested higher risks of upper respiratory tract infection (RR = 1.61, 95% CI 1.11–2.35), herpes zoster (RR = 3.88, 95% CI 1.70–8.89), and bronchitis (RR = 2.14, 95% CI 1.29–3.55), whereas infusion-related reactions did not differ significantly (RR = 1.22, 95% CI 0.62–2.40) (**Supplementary Figure S1**).

Other protocol-specified outcomes were handled according to data availability. LLDAS was pooled as a supportive secondary outcome where extractable data were available, whereas DORIS remission was summarized narratively because the available evidence came mainly from overlapping or *post hoc* sources. Deaths and injection-site reactions were sparse or route-specific and were therefore summarized narratively rather than treated as primary pooled endpoints.

### Supplementary and narrative results

#### Supplementary reports not included in the primary quantitative synthesis

Seven supplementary reports provided narrative evidence on organ-domain and mucocutaneous outcomes (45, 46), flare-related outcomes (47), stringent composite endpoints (43), remission and longer-term disease control (48), regional subgroup findings (49), and SLEDAI-2K item timing (50). Because these reports overlapped with parent trials or selected extension cohorts, they contextualized findings rather than adding independent participants to the meta-analysis. The characteristics, parent trials, and reasons for not treating these reports as independent studies are detailed in online **Supplementary Table S2**.

#### Subcutaneous anifrolumab evidence

TULIP-SC evaluated SC anifrolumab 120 mg weekly (27). BICLA, glucocorticoid tapering, and *post hoc* CLASI-50 outcomes generally favored anifrolumab, whereas annualized flare rate was only numerically lower, while DORIS remission and LLDAS were supportive. Safety observations, including herpes zoster, injection-site reactions, and one death from sepsis that was not judged to be treatment-related, remain limited by the single-study design.

### Sensitivity analyses

Sensitivity analyses assessed model choice using fixed-effect pooling, additionally evaluated Week 12 CLASI-A50 and SRI-4 after excluding TULIP-1, examined the influence of the registry-only AZALEA Asian phase III trial (NCT04931563) on sustained oral glucocorticoid reduction, BICLA response, and annualized flare rate, and used modified HKSJ analyses for outcomes with substantial heterogeneity and a small number of contributing studies.

Fixed-effect sensitivity analyses were broadly consistent in direction with the main random-effects analyses, including CLASI-A50 (RR 1.49, 95% CI 1.07–2.07), sustained oral glucocorticoid reduction (RR 1.55, 95% CI 1.31–1.84), BICLA (RR 1.65, 95% CI 1.43–1.92), SRI-4 (RR 1.40, 95% CI 1.23–1.59), and annualized flare rate (rate ratio 0.68, 95% CI 0.55–0.85) (**Supplementary Figures S3**–**S7**). The primary interpretation remained based on random-effects estimates. Exploratory analyses of Week 12 CLASI-A50 and SRI-4 after excluding TULIP-1 also preserved the favorable direction of effect (**Supplementary Figures S2A, B**). After excluding NCT04931563, the favorable effects were preserved for sustained oral glucocorticoid reduction (RR = 1.62, 95% CI 1.21–2.17; I² = 36.8%), BICLA response (RR = 1.60, 95% CI 1.30–1.97; I² = 26.1%), and annualized flare rate (rate ratio = 0.75, 95% CI 0.59–0.95; I² = 0.0%) (**Supplementary Figure S8**).

Modified HKSJ sensitivity analyses produced wider confidence intervals. For SRI-4 (RR 1.39, 95% CI 0.86–2.25), LLDAS (RR 1.80, 95% CI 0.12–26.77), and annualized flare rate (rate ratio 0.65, 95% CI 0.29–1.45), the confidence intervals crossed the null, indicating that the statistical significance of the primary random-effects estimates was not robust to the modified HKSJ approach. For LLDAS, this HKSJ sensitivity analysis should also be read in light of the exploratory source structure, because one contributing estimate was a pooled TULIP-1/TULIP-2 *post hoc* source rather than a separately extractable independent trial estimate. For serious adverse events (RR 0.90, 95% CI 0.39–2.06) and discontinuations due to adverse events (RR 0.91, 95% CI 0.17–4.84), which were already non-significant in the primary analyses, the modified HKSJ analyses further widened the confidence intervals, reflecting greater uncertainty (**Supplementary Table S6**).

### Certainty of evidence

GRADE certainty is detailed in online **Supplementary Table S5**. Certainty was high for glucocorticoid dose reduction and BICLA response, moderate for CLASI-A50, low for annualized flare rate, SRI-4, and serious adverse events, and very low for discontinuations due to adverse events. Downgrading was driven mainly by limited CLASI-A50 trials, heterogeneity for SRI-4 and safety outcomes, moderate heterogeneity for annualized flare rate, and imprecision for safety endpoints. The exclusion sensitivity analyses did not materially change the interpretation of sustained oral glucocorticoid reduction, BICLA response, or annualized flare rate, because the effect direction and statistical significance were preserved after excluding NCT04931563. The HKSJ sensitivity results supported a more cautious interpretation of heterogeneous secondary outcomes because their adjusted confidence intervals crossed the null.

## Discussion

### Principal findings

This review provides a clinically focused synthesis of randomized evidence for intravenous anifrolumab 300 mg every 4 weeks in SLE. The principal findings were that IV anifrolumab improved Week 52 CLASI-A50 response among patients with baseline CLASI-A ≥10 (23, 25) and increased sustained oral glucocorticoid reduction among patients receiving oral glucocorticoids at ≥10 mg/day at baseline (23, 25, 26, 28). BICLA response provided consistent supportive evidence of improvement in overall disease activity, whereas SRI-4, LLDAS attainment, and annualized flare rate were less certain because of heterogeneity or the limited number of contributing trials. The pooled safety analyses showed a modest increase in any adverse events and selected adverse events of clinical interest, but serious adverse events and discontinuations due to adverse events did not differ significantly and were imprecise. The primary conclusions therefore remain anchored in route-specific pooled evidence for the IV regimen and in the prespecified populations applicable to the two principal clinical outcomes. Modified HKSJ sensitivity analyses further indicated that the apparent statistical significance of several heterogeneous secondary outcomes was not robust to small-study random-effects uncertainty.

### Clinical interpretation of cutaneous and glucocorticoid-sparing outcomes

Cutaneous involvement and prolonged glucocorticoid exposure are distinct but related treatment concerns in SLE. The CLASI-A50 finding supports benefit for active inflammatory skin disease and applies most directly to patients with baseline CLASI-A ≥10, rather than to patients with absent or mild cutaneous activity (23, 25). CLASI-A50 reflects improvement in cutaneous activity rather than reversal of established damage, such as scarring or dyspigmentation. The exploratory Week 12 analysis provides supportive early-response information but only indirect information on sustained Week 52 cutaneous benefit. The primary Week 52 cutaneous finding should therefore be interpreted as two-trial, source-defined subgroup evidence; the TULIP-2 Week 12 result supports early cutaneous improvement but cannot substitute for unavailable Week 52 responder data.

The glucocorticoid-sparing evidence was more precise and was derived from four IV trials, but this endpoint applied only to patients receiving at least 10 mg/day at baseline and reflected protocol-defined tapering to ≤7.5 mg/day with maintenance from Weeks 40 through Week 52 (23, 25, 26, 28). This finding remains limited to protocol-defined tapering among eligible baseline glucocorticoid users in the included trials. The exclusion sensitivity analyses support the conclusion that the favorable findings for sustained oral glucocorticoid reduction, BICLA response, and annualized flare rate were not dependent on the registry-only Asian phase III trial. Tapering remains a clinical process guided by disease activity, flare history, organ involvement, and patient preference. Whether cutaneous improvement contributed to glucocorticoid reduction remains unresolved.

The crude event-count proportions are provided only to give clinical context for the pooled relative effects. Based on directly reported arm-level event counts, 58.8% of patients receiving anifrolumab achieved Week 52 CLASI-A50 compared with 38.8% receiving placebo, corresponding to a descriptive absolute difference of approximately 20 percentage points. Sustained glucocorticoid reduction was achieved by 54.1% and 34.6% of patients, respectively, an absolute difference of approximately 19.5 percentage points. These values are unweighted descriptive absolute differences based on crude event counts; they do not account for trial-specific weighting and should not be interpreted as pooled effect estimates or as substitutes for the formally pooled RRs. They also apply only to the corresponding prespecified baseline subgroups.

### Composite disease activity and flare outcomes

BICLA findings were relatively consistent and support a broader effect of anifrolumab on overall disease activity. By contrast, SRI-4 showed substantial heterogeneity. The reduction in heterogeneity after exclusion of TULIP-1 suggests an important trial-level source of inconsistency, but this exploratory analysis does not replace the prespecified all-trial analysis. Differences between BICLA and SRI-4 are not necessarily contradictory, because these composite endpoints capture different response definitions and treatment-failure rules. Flare-rate findings also favored anifrolumab but were based on few trials and moderate heterogeneity. LLDAS also favored anifrolumab, but it relied on MUSE plus a pooled TULIP-1/TULIP-2 *post hoc* source and should be regarded as exploratory supportive evidence rather than a conventional independent trial-level synthesis. In modified HKSJ sensitivity analyses, SRI-4, LLDAS, and flare-rate intervals crossed the null despite directionally favorable point estimates, so these outcomes should be considered supportive and hypothesis-strengthening rather than definitive.

### Safety findings and clinical monitoring

The non-significant serious adverse event and discontinuation estimates should be interpreted cautiously because confidence intervals were wide, events were sparse, heterogeneity was substantial, and the analyses were not powered to exclude clinically meaningful safety risks. Modified HKSJ analyses widened the serious adverse event and discontinuation intervals further, so these pooled estimates should not be interpreted as excluding clinically important safety differences.

Any adverse events and selected adverse events of clinical interest were more frequent in pooled or supplementary analyses, including upper respiratory tract infection, herpes zoster, and bronchitis, but these outcomes do not capture the complete safety profile, including serious infections, injection-site reactions, uncommon events, and longer-term safety. Clinical use therefore requires patient-level risk assessment, vaccination review when appropriate, infection surveillance, and careful monitoring during glucocorticoid tapering.

### Interpretation of subcutaneous evidence

Evidence for subcutaneous anifrolumab remains route-specific and exploratory. TULIP-SC provides useful single-trial information for the 120 mg once-weekly regimen, with several outcomes favoring anifrolumab (27). These data remain separate from the IV evidence, with clinical, pharmacokinetic, and safety equivalence still unproven. Potential advantages of SC administration, including convenience, reduced infusion burden, adherence, and patient preference, remain clinically relevant but require confirmation in additional route-specific and comparative studies. Future randomized head-to-head trials directly comparing IV and SC anifrolumab are needed to determine whether SC administration provides comparable efficacy, safety, pharmacokinetics, adherence, patient preference, and treatment burden.

### Clinical implications and patient selection

These findings support consideration of IV anifrolumab for selected patients with moderate-to-severe SLE who resemble the trial populations, particularly those with active cutaneous involvement, a clinically important need to reduce ongoing oral glucocorticoid exposure, or both. The results are most directly applicable to patients with baseline CLASI-A ≥10 for the cutaneous outcome (23, 25) and to those receiving at least 10 mg/day of oral glucocorticoids for the tapering outcome (23, 25, 26, 28). Beyond these groups, applicability is limited for patients without active skin disease, those receiving lower doses of or no glucocorticoids at baseline, or those whose main treatment objective concerns a disease domain not represented by these analyses. Applicability is also uncertain for patients with severe active lupus nephritis or severe neuropsychiatric SLE, who were generally excluded from pivotal trials. Within these boundaries, this synthesis may support shared decision-making by clarifying both potential benefits and the populations to whom they most directly apply.

### Comparison with previous evidence and contribution of this review

Previous evidence syntheses have addressed anifrolumab from different perspectives. The earlier meta-analysis by Lee et al. evaluated CLASI response and glucocorticoid reduction, but it included only the limited trial evidence available at that time and therefore did not incorporate subsequently reported randomized studies (51). Liu et al. focused primarily on safety and highlighted infection-related signals (22), whereas Mahmoud et al. assessed efficacy and safety across multiple organ domains, with cutaneous response and glucocorticoid tapering included within a broader set of outcomes rather than jointly prioritized as the principal clinical questions (29). Ding et al. compared anifrolumab with other biologic therapies using a network meta-analysis, addressing a different comparative question (30). Sciascia et al. examined glucocorticoid-sparing effects across biologic agents in a scoping review rather than conducting an anifrolumab-specific quantitative synthesis (2).

The present review extends this evidence by incorporating publicly available results from the Asian phase III trial (28) and by distinguishing five independent trials from 12 eligible reports. It avoids double-counting overlapping reports, separates intravenous and subcutaneous evidence, and restricts the primary quantitative synthesis to the approved intravenous regimen. Importantly, cutaneous response and sustained glucocorticoid reduction were interpreted within their respective prespecified baseline populations rather than generalized to the overall SLE population. The use of RoB 2 and GRADE further clarifies the risk of bias, certainty, applicability, and limitations of each pooled estimate (39–41).

These certainty ratings should be interpreted alongside clinical applicability. High-certainty evidence does not imply that the evidence is without limitations; rather, it indicates greater confidence in the estimated effect within the specific populations, regimens, and outcome definitions evaluated. For CLASI-A50, evidence was rated moderate because only two trials contributed, limiting precision and generalizability. Safety outcomes were rated low or very low because of few events, substantial heterogeneity, and wide confidence intervals. Thus, certainty of evidence and applicability to individual patients should be considered related but distinct dimensions.

### Strengths, limitations, and future research

The strengths of this review include its prespecified clinical focus, restriction of the primary quantitative synthesis to the approved IV regimen, separate handling of independent trials and overlapping reports, use of directly reported arm-level event counts, route-specific handling of SC evidence, and outcome-level assessment using RoB 2 and GRADE (39–41). A further strength is that the two principal outcomes were interpreted within their corresponding prespecified baseline populations—patients with baseline CLASI-A ≥10 for CLASI-A50 and those receiving oral glucocorticoids at ≥10 mg/day at baseline for sustained glucocorticoid reduction—rather than being presented as broadly applicable to the overall SLE population. We also added HKSJ sensitivity analyses to directly address small-study random-effects uncertainty for heterogeneous outcomes.

Several limitations should be acknowledged. The principal outcomes were derived from selected baseline subgroups: CLASI-A50 from patients with baseline CLASI-A ≥10 and glucocorticoid reduction from patients receiving at least 10 mg/day of glucocorticoids at baseline, which limits their direct applicability to the broader SLE population.

The pivotal evidence does not directly address patients with severe active lupus nephritis or severe neuropsychiatric SLE. In addition, the Asian phase III trial was available only through registry results rather than as a peer-reviewed full-text report, limiting appraisal of randomization procedures, missing-data handling, and reporting completeness (28). However, excluding this trial in sensitivity analyses did not materially alter the estimates for sustained oral glucocorticoid reduction, BICLA response, or annualized flare rate.

Several protocol-specified outcomes required adaptation because of data availability. Continuous changes in CLASI-A scores could not be consistently pooled, and the Week 52 CLASI-A50 analysis was limited to the source-defined baseline CLASI-A ≥10 subgroup for which extractable responder data were available. DORIS remission was not consistently available in an extractable form across independent eligible trials. A further limitation is that some treat-to-target outcomes were available primarily from pooled or *post hoc* TULIP-derived reports. Therefore, DORIS was not meta-analyzed, and the LLDAS estimate was treated as exploratory rather than as a conventional independent trial-level synthesis. Deaths were summarized narratively because of sparse events, whereas injection-site reactions were analyzed separately because they were specific to the subcutaneous route and were not suitable for pooling with intravenous safety data.

Statistical limitations include the small number of trials contributing to individual syntheses, the inability to formally assess funnel-plot asymmetry, and heterogeneity or imprecision in several secondary and safety outcomes. Modified HKSJ sensitivity analyses addressed uncertainty for outcomes with substantial heterogeneity, but precision remained limited by the small evidence base, heterogeneity, and sparse events.

Future studies should standardize cutaneous response thresholds, assessment time points, and glucocorticoid-tapering definitions; report cumulative glucocorticoid exposure; and place greater emphasis on patient-reported skin outcomes, quality of life, irreversible cutaneous damage, long-term organ damage, and real-world tapering feasibility. Larger and longer studies are needed to characterize infections, herpes zoster, and uncommon adverse events. Randomized head-to-head trials comparing IV and SC anifrolumab should also evaluate route-specific efficacy, safety, pharmacokinetics, adherence, patient preference, and treatment burden.

## Conclusion

This systematic review and meta-analysis supports IV anifrolumab 300 mg every 4 weeks plus standard therapy for improving Week 52 CLASI-A50 response among patients with baseline CLASI-A ≥10, although this primary cutaneous finding was based on two trials, and for facilitating sustained glucocorticoid reduction among patients receiving oral glucocorticoids at ≥10 mg/day at baseline. BICLA remained a consistent supportive outcome; SRI-4 and flare outcomes were directionally favorable but heterogeneous, while LLDAS provided only exploratory supportive evidence because it relied partly on a pooled TULIP-1/TULIP-2 *post hoc* source. HKSJ sensitivity analyses reinforced uncertainty for these secondary outcomes. Any adverse events and selected infection-related events were more frequent, whereas pooled serious adverse event and discontinuation outcomes showed no statistically significant difference. Evidence for SC administration remains limited to a single randomized trial. Overall, anifrolumab may be a clinically meaningful option for selected patients with moderate-to-severe SLE, especially those with active cutaneous involvement or a need for glucocorticoid reduction.

## Data Availability

The original contributions presented in the study are included in the article/**Supplementary Material**. Further inquiries can be directed to the corresponding author.

## Supplementary material

Supplementary Figure 1 Selected adverse events of clinical interest. **(A)** Upper respiratory tract infection. **(B)** Herpes zoster. **(C)** Bronchitis. **(D)** Infusion-related reactions.

Supplementary Figure 2 Exploratory week 12 CLASI-A50 and SRI-4 excluding TULIP-1. **(A)** CLASI-A50 response at week 12. **(B)** SRI-4 response after excluding TULIP-1.

Supplementary Figure 3 Primary efficacy outcomes, fixed-effect sensitivity analysis. **(A)** CLASI-A50 response at week 52. **(B)** Sustained oral glucocorticoid dose reduction.

Supplementary Figure 4 Secondary efficacy outcomes, fixed-effect sensitivity analysis. **(A)** BICLA response at week 52. **(B)** SRI-4 response at Week 52. **(C)** LLDAS attainment at Week 52. **(D)** Annualized flare rate.

Supplementary Figure 5 Safety outcomes, fixed-effect sensitivity analysis. **(A)** Any adverse events. **(B)** Serious adverse events. **(C)** Adverse events leading to discontinuation.

Supplementary Figure 6 Selected adverse events of clinical interest, fixed-effect sensitivity analysis. **(A)** Upper respiratory tract infection. **(B)** Herpes zoster. **(C)** Bronchitis. **(D)** Infusion-related reactions.

Supplementary Figure 7 Exploratory week 12 CLASI-A50 and SRI-4 excluding TULIP-1, fixed-effect sensitivity analysis. **(A)** CLASI-A50 response at Week 12. **(B)** SRI-4 response after excluding TULIP-1.

Supplementary Figure 8 Sensitivity analyses excluding the AZALEA Asian phase III trial (NCT04931563), random-effects models. **(A)** Sustained oral glucocorticoid dose reduction at Week 52. **(B)** BICLA response at Week 52. **(C)** Annualized flare rate.
