# Supplementary material for: Cutaneous efficacy and glucocorticoid-sparing effects of anifrolumab in systemic lupus erythematosus: a systematic review and meta-analysis of randomized controlled trials
Source: Front Immunol. 2026 Sep 30;17:1910969. doi: 10.3389/fimmu.2026.1910969 (PMC13662125; doi:10.3389/fimmu.2026.1910969)
Supplement: Supplementary file 1 [file Table1.docx]

**Cutaneous Efficacy and Glucocorticoid-Sparing Effects of Anifrolumab in Systemic Lupus Erythematosus: A Systematic Review and Meta-analysis of Randomised Controlled Trials**

**Supplementary data**

This supplementary file provides the complete search strategies, supplementary reports, risk-of-bias assessments, outcome definitions and data extraction rules, GRADE certainty assessment, and sensitivity analyses.

**Contents**

**1. Search strategies ........................................................ 2**

**Supplementary Table S1. Search strategies used for each database .................................. 2**

**2. Supplementary reports ........................................................ 3**

**Supplementary Table S2. Supplementary reports not treated as independent studies in the primary quantitative synthesis .................................. 3**

**3. Risk-of-bias assessment ........................................................ 4**

**Supplementary Table S3. Risk of bias assessment of included trials .................................. 4**

**4. Outcome definitions and data extraction rules ........................................................ 5**

**Supplementary Table S4. Definitions of outcomes and data extraction rules used in the meta-analysis .................................. 5**

**5. GRADE certainty of evidence ........................................................ 6**

**Supplementary Table S5. Summary of Findings and GRADE certainty of evidence assessment .................................. 6**

**6. Modified HKSJ sensitivity analyses ........................................................ 8**

**Supplementary Table S6. Modified Hartung-Knapp-Sidik-Jonkman sensitivity analyses for outcomes with substantial heterogeneity .................................. 8**

**7. Protocol deviations from PROSPERO ........................................................ 9**

**Supplementary Table S7. Deviations from the PROSPERO protocol and rationale .................................. 9**

**Supplementary Table S1. Search strategies used for each database**

Searches were conducted from January 1, 2011 to April 20, 2026 for all listed databases and trial-registry sources.

| **Database / registry** | **Complete search strategy** | **Date searched** | **Records identified** |
| --- | --- | --- | --- |
| PubMed | ("Lupus Erythematosus, Systemic"[Mesh] OR "systemic lupus erythematosus"[Title/Abstract] OR "lupus erythematosus, systemic"[Title/Abstract] OR SLE[Title/Abstract] OR "Libman-Sacks Disease"[Title/Abstract])  AND  ("anifrolumab"[Supplementary Concept] OR anifrolumab[Title/Abstract] OR "MEDI-546"[Title/Abstract] OR MEDI546[Title/Abstract] OR Saphnelo[Title/Abstract])  AND  ("Randomized Controlled Trial"[Publication Type] OR "Controlled Clinical Trial"[Publication Type] OR randomized[Title/Abstract] OR randomised[Title/Abstract] OR randomly[Title/Abstract] OR placebo[Title/Abstract] OR trial[Title/Abstract])  AND  ("2011/01/01"[Date - Publication] : "2026/04/20"[Date - Publication]) | April 20, 2026 | 116 |
| Embase | ('systemic lupus erythematosus'/exp OR 'systemic lupus erythematosus':ti,ab,kw OR 'lupus erythematosus systemic':ti,ab,kw OR sle:ti,ab,kw OR 'libman sacks disease':ti,ab,kw) AND ('anifrolumab'/exp OR anifrolumab:ti,ab,kw OR 'medi-546':ti,ab,kw OR medi546:ti,ab,kw OR saphnelo:ti,ab,kw) AND ('randomized controlled trial'/exp OR 'controlled clinical trial'/exp OR random*:ti,ab,kw OR placebo*:ti,ab,kw OR trial*:ti,ab,kw OR 'double blind':ti,ab,kw OR 'single blind':ti,ab,kw) AND [2011-2026]/py AND [01-01-2011]/sd NOT [21-04-2026]/sd | April 20, 2026 | 487 |
| Web of Science | TS=(  ("systemic lupus erythematosus" OR "lupus erythematosus systemic" OR SLE OR "Libman-Sacks disease" OR "Libman Sacks disease")  AND  (anifrolumab OR "MEDI-546" OR MEDI546 OR Saphnelo)  AND  (random* OR placebo* OR trial* OR "double blind" OR "single blind" OR "controlled trial")  )  AND PY=(2011-2026) | April 20, 2026 | 273 |
| Cochrane Library | #1 "systemic lupus erythematosus":ti,ab,kw OR "lupus erythematosus systemic":ti,ab,kw OR SLE:ti,ab,kw OR "Libman-Sacks disease":ti,ab,kw OR "Libman Sacks disease":ti,ab,kw  #2 anifrolumab:ti,ab,kw OR "MEDI-546":ti,ab,kw OR MEDI546:ti,ab,kw OR Saphnelo:ti,ab,kw  #3 #1 AND #2  Publication date: 2011-01-01 to 2026-04-20 | April 20, 2026 | 163 |
| ClinicalTrials.gov | Condition/disease: “Systemic Lupus Erythematosus”; Other terms: anifrolumab OR MEDI-546 OR Saphnelo; First posted: 2011-01-01 to 2026-04-20 | April 20, 2026 | 36 |

**Supplementary Table S2. Supplementary reports not treated as independent studies in the primary quantitative synthesis**

| **Report** | **Parent trial(s)** | **Report type** | **Reason not pooled independently** | **Use in this review** |
| --- | --- | --- | --- | --- |
| Morand et al., 2022, Efficacy of anifrolumab across organ domains in patients with moderate-to-severe systemic lupus erythematosus: a post-hoc analysis of pooled data from the TULIP-1 and TULIP-2 trials[44] | Pooled TULIP-1/TULIP-2 phase 3 trials | Post hoc pooled organ-domain analysis | Uses overlapping phase III TULIP trial populations already represented by their parent RCTs; organ-domain results would duplicate parent trial data if pooled independently | Describes organ-domain improvements |
| Furie et al., 2021, Anifrolumab reduces flare rates in patients with moderate to severe systemic lupus erythematosus[46] | TULIP-1 and TULIP-2 | Pooled post hoc flare analysis | Pooled analysis of the same phase III TULIP trial populations already represented by their parent RCTs | Adds narrative detail on flare reduction and flare control during glucocorticoid tapering |
| Werth et al., 2022, Improvement of individual mucocutaneous manifestations in patients with SLE treated with anifrolumab[45] | TULIP-1 and TULIP-2 | Pooled post hoc mucocutaneous analysis | Uses overlapping phase III TULIP trial populations and domain-level/post hoc mucocutaneous outcomes | Adds narrative detail on individual CLASI-A and mucocutaneous manifestation improvements |
| Isenberg et al., 2021, Novel stringent outcome measures applied to the phase 2 and 3 anifrolumab trials[47] | MUSE, TULIP-1, and TULIP-2 | Post hoc pooled analysis of stringent composite endpoints | Applies additional composite definitions to trial populations already included through parent RCTs | Adds narrative detail on stringent BICLA, dual-response, glucocorticoid-taper, and no-flare composite outcomes |
| Morand et al., 2025, LLDAS and remission attainment with anifrolumab treatment in patients with systemic lupus erythematosus: results from the TULIP and long-term extension randomised controlled trials[48] | TULIP-1/TULIP-2 and TULIP long-term extension | Long-term extension and post hoc LLDAS/DORIS remission analysis | Extension/remission analysis overlaps with original TULIP participants and includes selected patients entering long-term follow-up | Adds narrative detail on DORIS remission and longer-term disease-control outcomes |
| Tanaka et al., 2023, The efficacy and safety of anifrolumab in Japanese patients with SLE: TULIP-2 subanalysis[49] | TULIP-2 | Post hoc Japanese subgroup analysis | Subgroup analysis from an already included parent RCT; population is not independent of TULIP-2 | Adds narrative detail on consistency of efficacy and safety in Japanese patients |
| Vital et al., 2024, Timing of SLEDAI-2K item improvements during the first year of intravenous anifrolumab treatment of moderate to severe SLE[50] | TULIP-1 and TULIP-2 | Pooled post hoc SLEDAI-2K item and response-timing analysis | Pooled analysis of the same phase III TULIP trial populations with timing/item-level endpoints | Describes the timing of SLEDAI-2K item and organ-system improvements |

**Supplementary Table S3. Risk of bias assessment of included trials**

| **Study / Trial** | **D1** | **D2** | **D3** | **D4** | **D5** | **Overall risk of bias** |
| --- | --- | --- | --- | --- | --- | --- |
| MUSE (Furie 2017) | Low risk | Low risk | Low risk | Low risk | Some concerns | Some concerns |
| TULIP-1 (Furie 2019) | Low risk | Low risk | Low risk | Low risk | Low risk | Low risk |
| TULIP-2 (Morand 2020) | Low risk | Low risk | Low risk | Low risk | Low risk | Low risk |
| Asian phase III (NCT04931563) | Some concerns | Low risk | Some concerns | Low risk | Some concerns | Some concerns |
| TULIP-SC (Manzi 2026) | Low risk | Low risk | Low risk | Low risk | Some concerns | Some concerns |

D1, bias arising from the randomisation process; D2, bias due to deviations from intended interventions; D3, bias due to missing outcome data; D4, bias in measurement of the outcome; D5, bias in selection of the reported result. Risk-of-bias judgements were categorised as low risk, some concerns, or high risk according to the Cochrane RoB 2 tool.

Risk-of-bias judgements were made for the effect of assignment to intervention and were applied to the principal efficacy results used in this review. Where outcome-specific information differed, this was considered in the corresponding GRADE assessment.

**Supplementary Table S4. Definitions of outcomes and data extraction rules used in the meta-analysis**

| **Outcome** | **Definition** | **Analysis set** | **Preferred time point** | **Effect measure** | **Data extraction rule** |
| --- | --- | --- | --- | --- | --- |
| CLASI-A50 response | A ≥50% reduction from baseline in the Cutaneous Lupus Erythematosus Disease Area and Severity Index–Activity score. | Patients with baseline CLASI-A ≥10. | Week 52 where available. | RR | Arm-level event counts and denominators were extracted directly for intravenous anifrolumab 300 mg and placebo. When both event counts and percentages were reported, directly reported counts were used. Adjusted, weighted, model-based or posterior response rates were not converted into event counts. TULIP-SC reported CLASI-50 at Week 52 as a post hoc endpoint and was not included in the primary intravenous meta-analysis. |
| Sustained oral glucocorticoid reduction | Reduction of oral glucocorticoid dose to ≤7.5 mg/day by Week 40 and maintained through Week 52. | Patients receiving baseline oral glucocorticoid dose ≥10 mg/day. | Week 52. | RR | Extracted the number of patients achieving sustained reduction and the number of eligible patients at baseline. Data were extracted for anifrolumab 300 mg IV and placebo in the primary analysis. |
| BICLA response | BILAG-based Composite Lupus Assessment response, generally requiring improvement in baseline BILAG A/B scores without worsening in other BILAG organ systems, no worsening in SLEDAI-2K, no worsening in PGA, no treatment discontinuation, and no restricted medication use beyond protocol thresholds. | mITT population. | Week 52. | RR | Extracted responder counts and denominators for anifrolumab 300 mg IV and placebo. If studies reported amended or restricted-medication rules, the prespecified or primary analysis reported by the trial was preferentially used. |
| SRI-4 response | A ≥4-point reduction from baseline in SLEDAI-2K, no new BILAG A or ≥2 new BILAG B domain scores, and no worsening in PGA. | mITT population. | Week 52. | RR | Extracted responder counts and denominators for anifrolumab 300 mg IV and placebo. Week 52 data were preferentially used to maintain consistency across studies. |
| Annualized flare rate | Annualized rate of disease flares, commonly defined as ≥1 new BILAG-2004 A or ≥2 new BILAG-2004 B domain scores compared with the previous visit. | Trials reporting rate ratios. | Through Week 52. | Rate ratio | Extracted reported rate ratios and 95% CIs. The natural logarithm of the rate ratio and its standard error were calculated and pooled using the generic inverse variance method. This outcome was not analyzed using event/total data. |
| Serious adverse events | Any serious adverse event reported during the treatment or double-blind study period. | Safety population. | Through Week 52 or during the double-blind treatment period. | RR | Extracted the number of patients with at least one serious adverse event and the safety population denominator for each group. |
| Adverse events leading to treatment discontinuation | Any adverse event leading to discontinuation of investigational product or study treatment. | Safety population. | Through Week 52 or during the double-blind treatment period. | RR | Extracted the number of patients with adverse events leading to treatment discontinuation and the safety population denominator for each group. |

RR, risk ratio; CI, confidence interval; IV, intravenous; SC, subcutaneous; mITT, modified intention-to-treat; OCS, oral corticosteroid; CLASI-A, Cutaneous Lupus Erythematosus Disease Area and Severity Index–Activity; BICLA, British Isles Lupus Assessment Group-based Composite Lupus Assessment; BILAG, British Isles Lupus Assessment Group; SLEDAI-2K, Systemic Lupus Erythematosus Disease Activity Index 2000; PGA, Physician’s Global Assessment.

**Supplementary Table S5. Summary of Findings and GRADE certainty of evidence assessment**

Author(s): Jianing Wang, Feiyin Liu et al.

Question: Anifrolumab 300 mg IV every 4 weeks plus standard therapy compared to placebo plus standard therapy for adult patients with active systemic lupus erythematosus.

Setting: Multicenter randomised, double-blind, placebo-controlled phase IIb and phase III clinical trials evaluating IV anifrolumab 300 mg every 4 weeks plus standard therapy in adult patients with active systemic lupus erythematosus; four IV 300 mg q4w randomised trials contributed to the quantitative evidence profile.

Bibliography: Furie et al. 2017 (MUSE); Furie et al. 2019 (TULIP-1); Morand et al. 2020 (TULIP-2); ClinicalTrials.gov/NCT04931563 registry results for the Asian phase III trial [28].

CI, confidence interval; RR, risk ratio. Both groups received background standard therapy.

| **No. of studies** | **Study design** | **Risk of bias** | **Inconsistency** | **Indirectness** | **Imprecision** | **Other considerations** | **Anifrolumab** | **Placebo** | **Relative effect (95% CI)** | **Absolute effect (95% CI)** | **Certainty** | **Importance** |
| --- | --- | --- | --- | --- | --- | --- | --- | --- | --- | --- | --- | --- |
| **CLASI-A50 response at Week 52 (follow-up: mean 52 weeks; assessed with: Proportion of patients achieving ≥50% reduction in CLASI activity score from baseline)** | | | | | | | | | | | | |
| 2 | randomised trials | not serious | not serious | not serious | seriousa | none | 50/85 (58.8%) | 31/80 (38.8%) | RR 1.52  (1.09 to 2.11) | 202 more per 1,000  (from 35 more to 430 more) | ⨁⨁⨁◯  Moderatea | CRITICAL |
| **Glucocorticoid tapering maintained from Week 40 to Week 52 (follow-up: mean 52 weeks; assessed with: Proportion of patients with baseline oral glucocorticoid dose ≥10 mg/day who achieved dose reduction to ≤7.5 mg/day and maintained it from Week 40 to Week 52)** | | | | | | | | | | | | |
| 4 | randomised trials | not serious | not serious | not serious | not serious | none | 183/338 (54.1%) | 118/341 (34.6%) | RR 1.56  (1.31 to 1.86) | 194 more per 1,000  (from 107 more to 298 more) | ⨁⨁⨁⨁  High | CRITICAL |
| **BICLA response at Week 52 (follow-up: mean 52 weeks; assessed with: Proportion of patients achieving a BICLA response)** | | | | | | | | | | | | |
| 4 | randomised trials | not serious | not serious | not serious | not serious | none | 290/597 (48.6%) | 178/605 (29.4%) | RR 1.65  (1.42 to 1.91) | 191 more per 1,000  (from 124 more to 268 more) | ⨁⨁⨁⨁  High | IMPORTANT |
| **SRI-4 response at Week 52 (follow-up: mean 52 weeks; assessed with: Proportion of patients achieving an SRI-4 response)** | | | | | | | | | | | | |
| 4 | randomised trials | not serious | seriousb | not serious | seriousc | none | 317/597 (53.1%) | 233/606 (38.4%) | RR 1.39  (1.04 to 1.87) | 150 more per 1,000  (from 15 more to 335 more) | ⨁⨁◯◯  Lowb,c | IMPORTANT |
| **Annualised flare rate through Week 52 (follow-up: mean 52 weeks; assessed with: Annualised flare rate during the 52-week treatment period; absolute effect expressed per 1,000 patient-years)** | | | | | | | | | | | | |
| 3 | randomised trials | not serious | seriousd | not serious | not serious | none | 498 participants | 504 participants | Rate ratio 0.65  (0.47 to 0.91)  [Annualised flare rate through Week 52] | 222 fewer flares per 1,000 patient-years (from 337 fewer to 57 fewer) | ⨁⨁⨁◯  Moderated | IMPORTANT |
| **Serious adverse events during the intervention/safety follow-up period (assessed with: Proportion of patients experiencing at least one serious adverse event)** | | | | | | | | | | | | |
| 4 | randomised trials | not serious | seriouse | not serious | seriousf | none | 86/597 (14.4%) | 97/605 (16.0%) | RR 0.90  (0.54 to 1.50) | 16 fewer per 1,000  (from 74 fewer to 80 more) | ⨁⨁◯◯  Lowe,f | CRITICAL |
| **Discontinuation of intervention because of adverse events during the intervention/safety follow-up period (assessed with: Proportion of patients discontinuing treatment due to adverse events)** | | | | | | | | | | | | |
| 4 | randomised trials | not serious | seriousg | not serious | very serioush | none | 24/597 (4.0%) | 28/605 (4.6%) | RR 0.91  (0.33 to 2.55) | 4 fewer per 1,000  (from 31 fewer to 72 more) | ⨁◯◯◯  Very lowg,h | IMPORTANT |

**Explanations**

a. Downgraded one level for imprecision because the main analysis included only two trials with a limited number of participants, and the lower bound of the 95% confidence interval was close to no effect.

b. Downgraded one level for inconsistency because substantial heterogeneity was observed across trials (I² = 80.8%), mainly driven by the divergent effect estimate in TULIP-1. Sensitivity analysis excluding TULIP-1 reduced heterogeneity and supported the direction of benefit, but the main analysis was retained for certainty assessment.

c. Downgraded one level for imprecision because the lower bound of the 95% confidence interval was close to the line of no effect, indicating that the true effect may range from minimal benefit to a larger benefit.

d. Downgraded one level for inconsistency because moderate heterogeneity was observed across trials (I² = 56.5%), although all effect estimates favoured anifrolumab.

e. Downgraded one level for inconsistency because substantial heterogeneity was observed across trials (I² = 70.4%), with the AZALEA trial showing an effect estimate in the opposite direction compared with the other trials.

f. Downgraded one level for imprecision because the 95% confidence interval crossed the line of no effect and included both a possible reduction and a possible increase in serious adverse events.

g. Downgraded one level for inconsistency because substantial heterogeneity was observed across trials (I² = 66.0%), with effect estimates varying in direction across studies.

h. Downgraded two levels for imprecision because the number of events was small and the 95% confidence interval was wide, crossing the line of no effect and including both a possible reduction and a possible increase in discontinuation due to adverse events.

**Supplementary Table S6. Modified Hartung-Knapp-Sidik-Jonkman sensitivity analyses for outcomes with substantial heterogeneity.**

DerSimonian-Laird random-effects estimates remain the primary analyses. Modified HKSJ estimates are presented as sensitivity analyses for outcomes with I2 greater than 50% or specifically highlighted by Reviewer 2.

For LLDAS, the HKSJ analysis used MUSE plus a pooled TULIP-1/TULIP-2 post hoc source and should be interpreted as exploratory supportive evidence.

| **Outcome** | **k** | **Main DL random-effects estimate** | **I2** | **tau-squared** | **Modified HKSJ estimate** | **Interpretation** |
| --- | --- | --- | --- | --- | --- | --- |
| SRI-4 response | 4 | RR 1.39 (1.04-1.87) | 80.8% | 0.0737 | RR 1.39 (0.86-2.25) | Direction favored anifrolumab, but the HKSJ interval crossed the null; statistical significance is sensitive to small-study random-effects uncertainty. |
| LLDAS attainment | 2 | RR 1.80 (1.19-2.73) | 57.2% | 0.0549 | RR 1.80 (0.12-26.77) | Direction favored anifrolumab, but with only two contributing sources the HKSJ interval was extremely wide; this outcome should remain supportive. |
| Annualized flare rate | 3 | Rate ratio 0.65 (0.47-0.91) | 56.5% | 0.0494 | Rate ratio 0.65 (0.29-1.45) | Direction favored anifrolumab, but the HKSJ interval crossed the null; inference should be framed as supportive rather than definitive. |
| Serious adverse events | 4 | RR 0.90 (0.54-1.50) | 70.4% | 0.1930 | RR 0.90 (0.39-2.06) | No clear increase or reduction was shown; HKSJ further widened the interval, reinforcing uncertainty. |
| AEs leading to discontinuation | 4 | RR 0.91 (0.33-2.55) | 66.0% | 0.7058 | RR 0.91 (0.17-4.84) | No clear increase or reduction was shown; sparse events and HKSJ adjustment produced a very imprecise interval. |

Abbreviations: AE, adverse event; CI, confidence interval; DL, DerSimonian-Laird; HKSJ, Hartung-Knapp-Sidik-Jonkman; I2, I-squared; LLDAS, Lupus Low Disease Activity State; RR, risk ratio.

Source note: SRI-4, annualized flare rate, serious adverse events, and AEs leading to discontinuation were calculated from the extraction workbook. LLDAS study-level counts were transcribed from current final Figure 5, panel C because no structured LLDAS rows were present in the extraction workbook.

**Supplementary Table S7 Protocol deviations and implementation clarifications relative to PROSPERO**

We thank the reviewer for highlighting the need to clarify how the final synthesis related to the PROSPERO protocol. We have revised the manuscript to make the relationship between the registered plan and the implemented analyses transparent. The main statistical model is now aligned with the protocol: random-effects models are used for the primary quantitative syntheses, with fixed-effect models presented as sensitivity analyses. Remaining differences mainly reflect outcome availability, reporting format, route-specific evidence, sparse events, or overlapping post hoc sources.

| **Protocol item** | **Registered plan** | **Implemented in revised manuscript** | **Classification** | **Rationale** | **Location in revised manuscript** |
| --- | --- | --- | --- | --- | --- |
| Statistical model | Random-effects models planned as the primary approach; fixed-effect models may be used in sensitivity analyses. | Random-effects models are used for all primary quantitative syntheses; fixed-effect results are presented only as sensitivity analyses. | No deviation / aligned after revision | This directly aligns the manuscript with the registered plan and avoids interpreting fixed-effect estimates as primary results. | Methods; Table 2; Figures 4-6; Supplementary Figures S3-S7 |
| Primary IV synthesis and SC evidence | Eligible anifrolumab randomized evidence was planned for synthesis. | The primary quantitative synthesis is restricted to IV anifrolumab 300 mg every 4 weeks; TULIP-SC is summarized narratively. | Implementation clarification | IV and SC regimens differ by route and dose, and TULIP-SC is a single route-specific trial; pooling it with IV trials could create clinical heterogeneity. | Methods; Results: TULIP-SC; Discussion |
| CLASI-A activity score change | CLASI-A activity score change was included as a planned cutaneous outcome. | Continuous CLASI-A change was not pooled; quantitative cutaneous synthesis used CLASI-A50 responder data. | Deviation due to data availability/reporting format | Continuous change data were not consistently reported in a form suitable for quantitative pooling across trials, whereas CLASI-A50 responder counts were extractable and clinically interpretable. | Methods: data extraction; Results: CLASI-A50; Limitations |
| Baseline CLASI-A subgroup | The protocol did not specify a baseline CLASI-A >=10 restriction. | Week 52 CLASI-A50 synthesis is restricted to patients with baseline CLASI-A >=10, as reported by source trials. | Implementation clarification | This was a source-defined eligibility subgroup for the extractable CLASI-A50 data, not a post hoc broadening of the target population. | Abstract; Methods; Results; Discussion |
| LLDAS | LLDAS was registered as a low disease activity outcome. | LLDAS is pooled as a supportive secondary outcome where extractable data are available. | No deviation / included with restricted interpretation | Available data allow a quantitative supportive analysis, but contributing sources are limited and heterogeneity is present. | Results; Table 2; Figure 5C |
| DORIS remission | DORIS remission was registered as a remission outcome. | DORIS remission is summarized narratively rather than pooled. | Deviation due to data availability/source overlap | Available DORIS evidence came mainly from overlapping, post hoc, or extension sources, so quantitative pooling as an independent endpoint was not appropriate. | Results: TULIP-SC/overlapping reports; Limitations |
| Infections, herpes zoster, URTI, bronchitis, infusion-related reactions | Selected adverse events, including infections, herpes zoster, upper respiratory tract infection, bronchitis, and infusion/injection-site reactions, were registered. | URTI, herpes zoster, bronchitis, and infusion-related reactions are summarized in supplementary random-effects analyses; fixed-effect versions are presented as sensitivity analyses. | Included as supplementary analyses | These outcomes are clinically important but secondary to the main safety endpoints; they are therefore presented as supplementary analyses rather than main pooled safety outcomes. | Results: selected adverse events; Supplementary Figure S1; Supplementary Figure S6 |
| Injection-site reactions | Infusion or injection-site reactions were registered. | Infusion-related reactions are pooled where available; injection-site reactions are summarized narratively. | Deviation due to sparse/route-specific data | Injection-site reactions are route-specific and sparse, especially because the primary quantitative synthesis focuses on IV trials. | Results: safety; TULIP-SC; Limitations |
| Deaths | Deaths were registered as a safety outcome. | Deaths are summarized narratively rather than pooled. | Deviation due to sparse events | Deaths were too sparse for a meaningful pooled estimate and were therefore handled narratively. | Results: TULIP-SC/safety; Limitations |
| Week 12 CLASI-A50 and SRI-4 excluding TULIP-1 | These analyses were not the primary registered analyses. | Week 12 CLASI-A50 is presented as exploratory/supportive; SRI-4 excluding TULIP-1 is presented as a sensitivity analysis. | Additional exploratory/sensitivity analyses | These analyses clarify timepoint availability and investigate heterogeneity without replacing the prespecified Week 52/random-effects main analyses. | Results; Sensitivity analyses; Supplementary Figure S2 |
